# Artificial Dentistry, Patents, &c.

**Published:** 1860-10

**Authors:** S. M. Shepherd


### ARTICLE VIII.

*Artificial Dentistry, Patents, &c.* By S. M. SHEPHERD, M. D., D. D. S.

IT may be truly said, "The world is on the wing." In every department of science and art the watchword is "Advance!" Wonderful things are constantly being brought to light, and the word is heard on almost every passing breeze—"See, here is something new!" Conspicuous in the array of great achievements, is found the subject on which I now propose to write. There is no science connected with the interests of the human family that has made such rapid strides within the last fifty years as has that of Dentistry. It is astonishing to think how small a space it occupied in the world at so recent a date as a half century ago. Indeed, it can scarcely be said to have existed as a science at that day. True, there were a few men in the world who professed to be dentists, and performed operations on the teeth, but they had no laws by which to govern them in their operations, and if they conferred benefit it was the result of accident rather than skill. There was no literature—probably a dozen books or less would have comprised everything that had ever been written on the subject, and these contained the most absurd ideas imaginable, as has been proved by more recent developments. I say it is strange that a science which now occupies so large a space in the world, and one that is so important to the comfort of mankind, so extensively known, too, and appreciated, should have been so little known only fifty years ago.

There is now scarcely a town in all the civilized world, in which there is not one or more local dentists, possessed of all the modern improvements. Books by the hundred, have been written on the subject, and men capable of adorning any profession have entered the ranks; and colleges have been erected and filled with able professors, purely for the purpose of teaching this branch of the healing art.

But it is not my purpose to write a history of the science in any of its branches; nor will I undertake to describe the progress of that particular branch which heads my article, though it would be a pleasing task with the proper material at hand, to give an account of artificial dentistry, from the first crude operation down to the present mode as practiced all over the land. I only wish to speak of it as it is at the present day. If all the achievements which have been wrought out in this department of the science, were suddenly blotted out from the world, what a "shaking there would be among the dry bones." Countless thousands are enjoying untold benefits as the results of labor and skill in this branch, and still, *advance* is the word. While all the branches of dental science have rapidly progressed, the artificial branch has outstripped them all. By patient labor, both of mind and body, the most astonishing results have been reached. A whole denture is now so adapted to the mouth as to elude the closest observation as to there being anything artificial about it; and to the wearer they so fulfill the wants of nature in this regard, as to cause him to forget that he has ever been deprived of his natural organs. One might think that reasonable minds ought to be satisfied with such results, and be content to "hold fast that whereunto they have attained," and cease to labor for still higher attainments. But not so; the attainment of one benefit seems only to sharpen the appetite of the pursuer for other and larger benefits. What the end will be cannot be foreseen, but it is a subject of deep interest, both to the profession and to the public.

There has been an "irrepressible" tendency on the part of *inventors*, to cover their inventions with letters patent; and an equally strong opposition to such a course on the part of a large portion of the profession, who think that the dignity of the profession is lowered by it. This I have regarded as unfortunate—looking at it purely in the light of an advancing science. Those who make valuable discoveries, contend, with much plausibility, that they cannot afford to give to the profession the result of their investigations, which has cost them time, labor and money; and they only wish a reasonable compensation, while the opposing parties contend that the dental profession ought to be on a level with the medical, or that it is and ought to be considered a cöordinate branch of the medical profession, and there are no letters patent in the medical profession. Without attempting to decide the merits of the controversy, I may be allowed to express the opinion that it is unfortunate for both parties, as well as for the profession as a whole. So long as inventors claim remuneration for their improvements, the improvements must be confined to that class of operators who tolerate patents; and by consequence the tariff on patents must be proportionably high, and that of itself is a bar to many who have no objection to the patents, but are unable to pay the price. Those who are opposed to patents shut themselves off from improvements that are known and acknowledged to be such, and thus not only deprive themselves of the advantages which would result from their use, but they deprive their patrons of benefits enjoyed by the patrons of others who may be less skillful than themselves. As a general thing, those who oppose patents are high-toned gentlemen, well educated, and occupy high positions in the profession, and could, if they would, give credit to any real improvement that may at any time be made. But they scorn to give countenance to anything, good or bad, that comes to them under letters patent. Now, if we could compel our laborious inventors to *give* us the result of their labors free of cost, then no doubt all would be willing to examine and profit by all the real improvements that might be offered. But this we cannot do, and since we cannot profit by the labor of others without paying a small fee for it, the interest of the profession as well as that of the public seems to demand that we should tolerate patents—at least in the artificial department. If countenance could be given to the good, it would become better. Men of skill would be able to bring to perfection the crude idea that is sometimes offered as perfect, when in fact it is very far from it. Indeed the very best improvements we have, in all departments of science, require to be worked by men of practical skill in order to their perfection; and there is probably no species of contrivance known that profits more by skillful working than artificial dentistry.

The great ends to be sought in adapting artificial teeth, are, first, healthfulness of the mouth. To this end, no diseased teeth or roots should be left in its preparation for plates. And secondly, a comfortable fit of plates, of whatever kind, to the mouth. This, of course, embraces a conformity to nature as near as possible, both in feeling and appearance. And thirdly, durability; which includes purity and strength, with proper instructions to the wearer in regard to accidents from carelessness. In regard to the first, every dentist has his difficulties. Some people who *must have teeth*, will not submit to the right preparation of the mouth for them. This is always a trouble to the dentist who has a right appreciation of his calling, because his highest object is to make the mouth healthy; and he knows he is contributing to make it otherwise when he covers with plates, unsound and unhealthy roots. But he cannot help it and ought not to be blamed for the result. The second point named, viz. a comfortable fit, has been more difficult to attain than almost any one thing upon which so much time and labor has been spent. For many years, gold was the only material known as a base for artificial teeth; and the only mode of preparation was that of rolling it out into plates to be cut and fitted to models between dies made of the softer metal. A *perfect fit* never could be obtained by this process, for several reasons. In the first place, the shrinkage of the metals rendered it impossible to get a perfect die; and in the second place, the die being softer than the gold plate, when the gold plate came to be forced down upon the die to give it form, the small prominences on the die always yielded more or less, which, of course, prevented the plate from taking the true shape. Many expedients were resorted to for the purpose of overcoming these difficulties, but none succeeded fully. A studied combination of metals for dies, with a view to hardness, so as to maintain shape under the hammer, and at the same time to cool without shrinkage, resulted in some benefit, but fell short of the objects. I know that plates were so constructed as that an approximation toward a fit was obtained ; but a *perfect fit never*. But the most annoying circumstance is the warpage of the plates, which always occurs to a greater or less extent in the process of soldering on the teeth. The fit of the plate is never, after soldering, what it was before. These, and various other difficulties met with in the working of gold plates, have led to the application of other substances to this purpose—of which Blandy's Alloy, by the cheoplastic process, and the Vulcanite, or hard rubber, are in the most extensive use. I have not sufficient acquaintance with the latter to speak of it definitely. It looks well, and seems to be capable of being perfectly fitted, and at the same time of filling all interstices, so as to render a piece cleanly. I have some doubt whether it will not become frail and disintegrate by use. This can only be determined by time.

With the *cheoplastic* process I am more familiar, I have used it in my practice now three years and a half, and can therefore give some points in its history as well as its present adaptation. I feared at first that the metal would corrode in the mouth, which if it did, of course would render it unfit for the purpose. But I had such assurances that it had not, so far, I was induced to try it. It possessed so many palpable advantages in other respects over any thing else I had seen, I was disposed to try it and take the risk. I soon found that I could not use the teeth prepared by the patentee for the process ; and if I could not have obtained a different tooth I should have been forced to give it up in the very beginning. But I conceived that a tooth might be made that would be better adapted, or at least that I could use to my own satisfaction, I therefore got up a model and sent to Messrs. Jones, White & McCurdy, of Philadelphia, and they declined to make it, on the ground that it was very expensive, and being a new thing it was doubtful whether it would ever pay. I then sent to Messrs. Orum & Armstrong of the same city, who also declined at first, but on reflection they concluded to do it. The result was that I got a tooth exactly suited to the process, according to my judgment. I have used these teeth exclusively from that time to the present, and I find them easy of application in all practicable cases. They are also strong—never separating from the metal without first breaking. The shape of my tooth enables me to present a perfect crown above the metal—inside as well as out—and to finish the surfaces of metal around the necks of the teeth in accordance with the shape of the natural gums better than any other tooth I have seen. When I obtained this tooth I thought, if the metal was pure enough to be worn in the mouth, I could make of it all I wanted. To insure the purity of the metal, I adopted the plan of gilding, by the galvanic process—for which purpose I ordered a small battery, with which to do my own gilding. I used for the purpose *pure gold*, and gilded thoroughly. Gilding was not claimed by the patentee as at all necessary; but a piece looked better, and I fancied it was better; and now, after a trial of three years and a half I am satisfied that I was right in that conjecture. I have put in only one piece from the beginning until now, so far as I recollect, without gilding, and that was temporary. In casting the plate for that piece the metal did not flow freely, and a hole was left in the plate about the size of a gold dollar, which required to be filled up, and it was done with a solder that was prepared by the patentee for such purposes. The piece was worn six or eight months, and a new piece was constructed to take the place of the first, and to be permanent. On examination of the first piece I found that corrosion had taken place on the patch composed of solder, and it was nearly destroyed—corrosion having gone quite through it. I had very few defects of this sort after the first, requiring to be patched ; but at the first they were common ; I finally learned however, how to cast perfect plates. But many of my first pieces had patches of solder in them, and this is the only one I ever saw destroyed by corrosion. I naturally inferred that they were protected by gilding. I suppose if this one had been well gilded I should not have seen any corrosion upon it. I have in my possession a whole upper set which required to be renewed after it had been worn twelve months, and though it was *lightly* gilded it retained its purity perfectly, and the eye cannot detect the least corrosion upon it. There were many advantages claimed by the patentee for this style of work over all others, all of which I have found, so far to be just, and after a trial of three and a half years, I prefer it decidedly to any other. It requires nicety of workmanship, but when *done up right*, it does seem to answer all necessary ends better than any thing else I know of. To ensure a perfect fit, I have no doubt that impressions taken in plaster are far more accurate than those taken in wax—especially for whole sets. Plaster fully represents the soft as well as the hard parts of the mouth, and when a plate is cast on a model that was taken from a plaster impression, I find that it rests firmly on the palatine arch and does not require pressure to *carry it home*—so to speak. There is nothing that gives more satisfaction, both to the dentist and his patient than a perfect fit; and this he may have infallibly by *casting* his plate on models taken from plaster impressions. I speak of this as a mode of inserting artificial pieces, with which I am acquainted; there may be other modes equally good. Whatever mode gives *entire* satisfaction to the dentist and his patient is good, and the dentist will not give it up, if he is a sensible man, unless something better presents itself. I am frequently called on by men who offer me new modes of putting up teeth, and when I tell them that I use the cheoplastic mode and like it, they express astonishment, and often say, "you are the only man I have met with that uses it." I suppose their object is to invalidate every other style that they may make sale of their own. My motto is in these matters, "prove all things and hold fast that which is good." If I could have this style of work a *little lighter*, I think I would like it a little better. In whole upper sets it would be an advantage to have it a little lighter, in some cases. For lower sets, or partial sets, the weight never troubles me; and I do not think it would be a whit better if it could be made as light as a cork.

I would therefore suggest to members of the profession whether it would not be better to deal more leniently with patents, and let them have a fair chance, so that if they have any thing good in them it may be brought out. I was constitutionally opposed to patents, but I found that I could not get what I wanted without them, and though it went against the grain to patronize them, I was compelled to do it or be the sufferer myself. I do not regret it. I would not to-day, for half my artificial practice, relinquish my right to the use of the cheoplastic process, and still I wish all other modes prosperity; and if any other mode ever gets to be better than cheoplasty, I shall only have to be convinced of it to adopt it.

The artificial branch of our profession is, as I have shown, an important branch, and one that has undergone more decided improvements than any other, and as I have stated before, it is to the interest both of the profession and the public, that enterprise in this department should be fostered.

